# Carbon fixation pathways across the bacterial and archaeal tree of life

**DOI:** 10.1093/pnasnexus/pgac226

**Published:** 2022-10-04

**Authors:** Alessandro N Garritano, Weizhi Song, Torsten Thomas

**Affiliations:** Centre for Marine Science and Innovation, School of Biological, Earth and Environmental Sciences, Faculty of Science, The University of New South Wales, Kensington, NSW 2052, Australia; Centre for Marine Science and Innovation, School of Biological, Earth and Environmental Sciences, Faculty of Science, The University of New South Wales, Kensington, NSW 2052, Australia; Centre for Marine Science and Innovation, School of Biological, Earth and Environmental Sciences, Faculty of Science, The University of New South Wales, Kensington, NSW 2052, Australia

**Keywords:** carbon fixation cycles, reductive tricarboxylic acid cycle, 3-hydroxypropionate bi-cycle, Calvin–Benson–Basshan cycle, 3-hydroxypropionate/4-hydroxybutyrate

## Abstract

Carbon fixation is a critical process for our planet; however, its distribution across the bacterial and archaeal domains of life has not been comprehensively studied. Here, we performed an analysis of 52,515 metagenome-assembled genomes and discover carbon fixation pathways in 1,007 bacteria and archaea. We reveal the genomic potential for carbon fixation through the reverse tricarboxylic acid cycle in previously unrecognized archaeal and bacterial phyla (i.e. *Thermoplasmatota* and *Elusimicrobiota*) and show that the 3-hydroxypropionate bi-cycle is not, as previously thought, restricted to the phylum *Chloroflexota*. The data also substantially expand the phylogenetic breadth for autotrophy through the dicarboxylate/4-hydroxybutyrate cycle and the Calvin–Benson–Bassham cycle. Finally, the genomic potential for carbon fixation through the 3-hydroxypropionate/4-hydroxybutyrate cycle, previously exclusively found in *Archaea*, was also detected in the *Bacteria*. Carbon fixation thus appears to be much more widespread than previously known, and this study lays the foundation to better understand the role of archaea and bacteria in global primary production and how they contribute to microbial carbon sinks.

Significance Statement Global carbon fixation is underpinned by at least seven different biochemical pathways, of which six are exclusively found in bacteria and archaea. Here, we provide a comprehensive picture on how these carbon fixation pathways are distributed across bacterial and archaeal domains of life and discover the unexpected occurrence of certain pathways in specific phylogenetic and taxonomic groups. These findings have significant implications as they show that a much greater diversity of bacteria and archaea have the potential to contribute to carbon fixation than previously known.

## Introduction

Autotrophic organisms are crucial for supporting life on Earth and are primary producers in all ecosystems. Both marine and terrestrial environments contribute equally to the rate of net primary production, and most of the global CO_2_ is thought to be fixed into organic matter through the Calvin–Benson–Bassham (CBB) cycle (1, 2). On land, this process is mainly carried out by eukaryotic plants, while in the oceans it is mainly driven by microscopic phytoplankton, including microalgae and cyanobacteria (3).

Although carbon fixation pathways (CFPs) other than the CBB cycle have been known for a long time, only recently has it been accepted that they also contribute significantly to global net primary production (4, 5). Besides the CBB cycle, there are six other known CFPs found in bacteria and archaea: the reverse tricarboxylic acid (rTCA) cycle, the 3-hydroxypropionate (3HP) bi-cycle, the 4-hydroxybutyrate/3-hydroxypropionate (4HB/3HP) cycle, the dicarboxylate/4-hydroxybutyrate (DC/4HB) cycle, the reductive acetyl-CoA pathway (Wood–Ljungdahl pathway—WLP), and the reductive glycine pathway (6). Some of these CFPs, such as the rTCA cycle, are found across several different bacterial and archaeal phyla, while others are thought to occur only in particular taxa (e.g. the 3HP bi-cycle in the phylum *Chloroflexota*) (7, 8).

Recent large-scale metagenomic studies have been able to uncover the genomic diversity of much of the bacterial and archaeal life on our planet (9–11). One of the most comprehensive studies was recently conducted by Nayfach et al., which describes 52,515 metagenome-assembled genomes (MAGs) that cover all major taxonomic groups (12). These resources now provide the opportunity to comprehensively describe the distribution of CFPs in the bacterial and archaeal domains of life.

Here, we use bioinformatics and a hand-curated analysis to describe the occurrence of CFPs in the *Archaea* and *Bacteria*. We show evidence for the previously unrecognized occurrence of the rTCA cycle in certain archaeal and bacterial phyla (*Thermoplasmatota* and *Elusimicrobiota*) as well as an expanded phylogenetic distribution of the 3HP bi-cycle, the DC/HB cycle, and CBB cycle. The HP/HB cycle, which was previously exclusively found in the *Archaea*, was here also detected in the *Bacteria* and found to be linked to photosynthesis in the genus *Luminiphilus*.

## Results and discussion

**Fig. 1.**
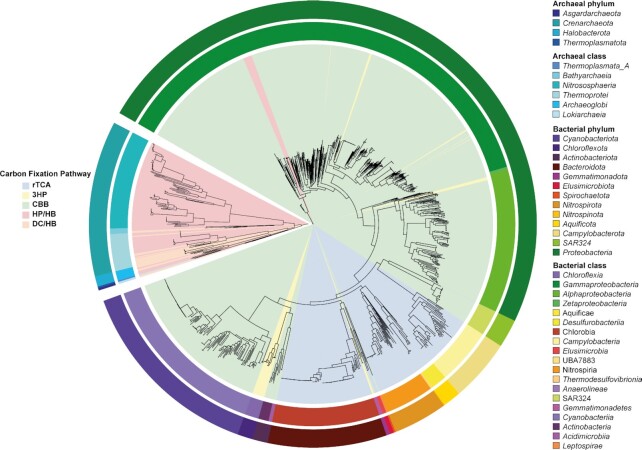
Phylogenetic tree representing the distribution of the different CFPs across the bacterial and archaeal domains of life in 1,007 MAGs. The outer ring is colored by the phylum, while the inner ring shows classes. Shading in the cycle shows the five different CFPs analyzed by the pipeline (see text for details).

In this study, we analyzed the distribution of five CFPs across a dataset of 52,515 MAGs that belong to the domains *Archaea* (5.8% of MAGs) and *Bacteria* (94.2% of MAGs), and contain 12,556 novel candidate species (12). CFPs were confidently identified in 1007 MAGs (Fig. 1) and 23 MAGs have more than one pathway [Table S5; see the “Material and methods” section for details on the identification process and the taxonomic classification using the Genome Taxonomy Database (GTDB) (13)]. As the WLP, the reverse oxidative TCA cycle, and the glycine reductive pathway can run in the oxidative direction with the same enzymes (i.e. in methane or acetate oxidizers) and have only reversible enzymatic reactions (14, 15), an accurate in-silico prediction is not possible and hence was not pursued here.

Energy-generating pathways related to the oxidation of 11 different compounds as well as the biosynthesis of (bacterio-)chlorophyll were also analyzed to understand how the organisms generate reductive power for carbon fixation. No correlation was found between a specific habitat from which the MAGs were recovered and the type of CFP we detected in the MAGs (Fig. S1). Specific observations on each CFP are presented and discussed in the following sections.

### Distribution of the reverse tricarboxylic acid cycle

The rTCA cycle, also known as the Arnon–Buchanan cycle, was first described in the green photosynthetic, anoxygenic bacterium *Chlorobium thiosulfatophilum* [phylum *Bacteroidota*, formerly phylum *Chlorobi* (16)] and most of its enzymes catalyze reversible reactions that are also used in the oxidative TCA cycle. However, the reduction of fumarate to succinate and the cleavage of citrate into acetyl-CoA and oxaloacetate reactions are considered to be irreversible (15, 17). The citrate cleavage can be catalyzed either by an ATP-dependent citrate lyase (ACL) (hereafter referred to as rTCA1, see Table 1) or by the sequential reactions of the ATP-dependent citryl-CoA synthetase (CCS) and the citryl-CoA lyase (CCL) (hereafter referred to as rTCA2, see Table 1). CO_2_ is fixed in the conversion of succinyl-CoA to 2-oxoglutarate as well as in the conversion of 2-oxoglutarate into isocitrate. Under low product-to-substrate ratios, the regular citrate lyase can also cleave citrate to support the rTCA (15); however, this cannot be bioinformatically distinguished from the oxidative version of the TCA and was therefore not considered in the current study. So far, this pathway has been shown to be restricted to the genus *Desulfurella*, but it is possible that other organisms known to express enzymes of the rTCA cycle and carrying citrate lyase genes might have the potential to use this cycle (15).

**Table 1.** Key enzymes, reference organisms (and accession numbers), and pathway completeness thresholds of the different CFPs.

**Autotrophic pathway**	**Key enzyme**	**Reference organism**	**Number of reactions in CFP**	**Expected completeness threshold (%)**	**Number of detected MAGs**
rTCA1	Citrate lyase A (ACLa)Citrate lyase B (ACLb)	*Chlorobium limicola* (16)(CP001097.1)	9	90	207
rTCA2	Citryl-CoA lyase (CCL)Citryl-CoA synthetase small (CCSs)Citryl-CoA synthetase large (CCSl)	*Hydrogenobacter thermophilus* (18)(CP002221.1)	12	73	10
3HP	Mesaconyl-CoA isomerase(TIGR04253)	*Chloroflexus auranticus* (19)(CP000909.1)	26	82	21
CBB	Phosphoribulokinase I (PRK1)Phosphoribulokinase II (PRK2)	*Cereibacter*(formerly*Rhodobacter*)*sphaeroides* (20)(CP001150.1)	13	90	616
HP/HB	4-Hydroxybutyrate dehydrogenase(HBD)	*Nitrosopumilus maritimus* (21)(CP000866.1)	16	90	135
DC/HB	4-Hydroxybutyrate dehydrogenase(HBD)	*Ignicoccus hospitalis* (22)(CP000816.1)	14	75	18

Citrate is cleaved into oxalacetate (OAA) and acetyl-CoA in a one-step reaction catalyzed by ACL in rTCA1. The rTCA2 pathway is a two-step catalysis, in which citrate is first cleaved into citryl-CoA by CCS and then citryl-CoA is cleaved into OAA and acetyl-CoA.

Consistent with the literature, we found genetic support for the rTCA1/2 cycles within a wide diversity of bacterial taxa within the phyla *Aquificota* (genera *Hydrogenobacter, Thermocrinis, Hydrogenobaculum, Persephonella, Sulfuridrogenibium*, and *Thermovibrio*), Bacteriodota (in particular the families *Chlorobiaceae* and *Chloroherpetonaceae*), *Campylobacterota* (specifically the class *Campylobacteria*; formerly *Epsilonproteobacteria*), and *Nitrospinota* (18, 23–25) (Table S1).

**Fig. 2.**
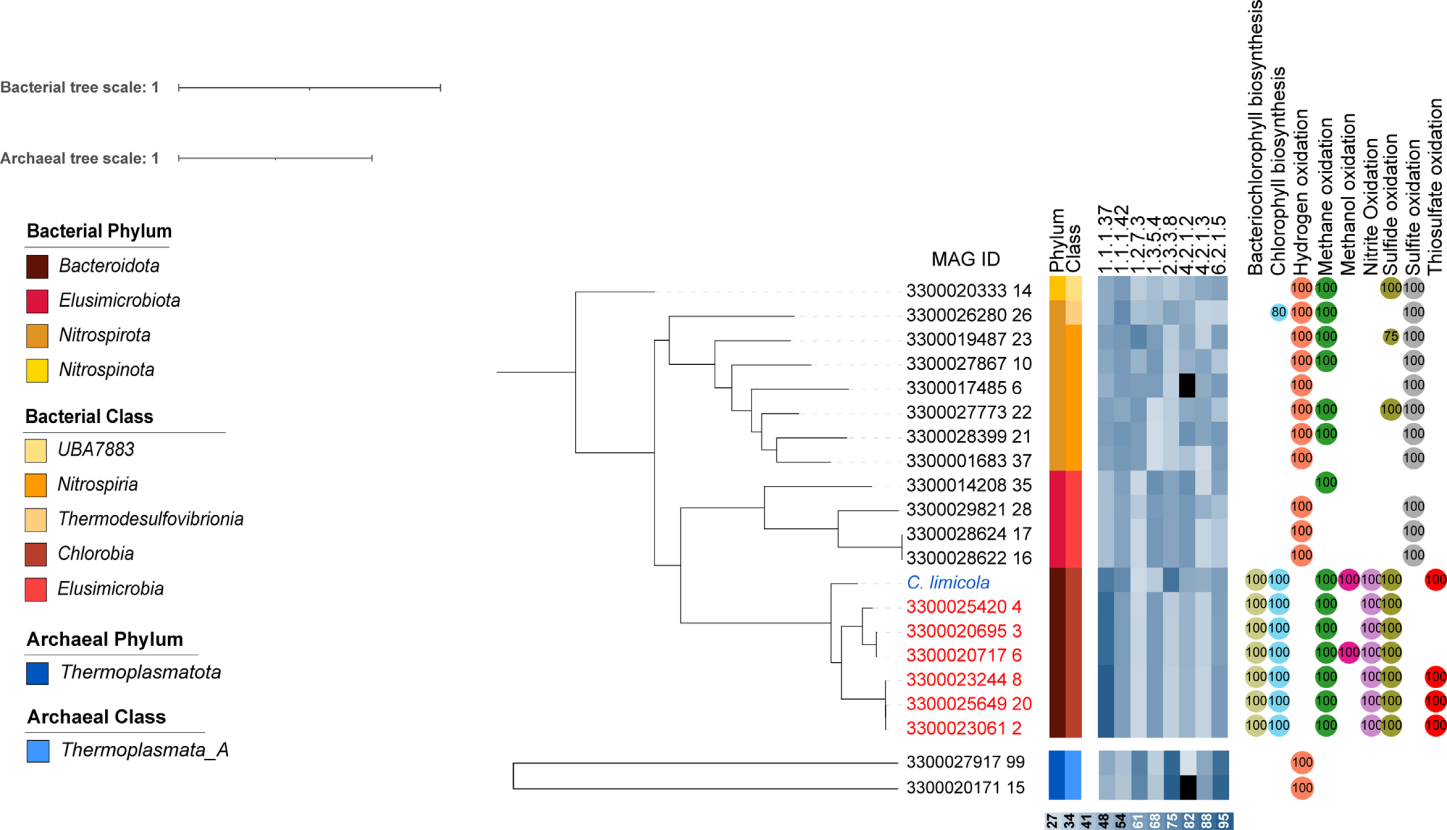
Phylogenetic tree showing the distribution of the rTCA cycle in novel bacterial and archaeal taxa and in known organisms from the family *Chlorobiaceae* (blue: reference genome in blue; red: MAGs assigned to the same family as the reference genome). Identity of the enzymes involved in the pathway against the UniRef database are shown in blue heat scale. 1.1.1.37: pyruvate-water dikinase; 1.1.1.42: isocitrate dehydrogenase; 1.2.7.3: 2-oxoglutarate-synthase; 1.3.5.4: phosphoenolpyruvate carboxylase; 2.3.3.8: ATP-citrate lyase; 4.2.1.2: fumarase; 4.2.1.3: aconitate hydratase; 6.2.1.5: succinyl-CoA synthetase. Numbers in circles refer to completeness of diagnostic pathways for energy generation. Black squares in the heatmap refers to missing enzymes.

In addition to these previous observed taxa, we found here that two archaeal MAGs (GEM ID 3300020171_15 and 3300027917_99) had the rTCA1 cycle with adjusted, observed completeness greater than 97% and the predicted capacity for hydrogen oxidation (Fig. 2).

The proposition of the rTCA1 cycle in the domain *Archaea* is not novel. Some evidence for the rTCA1 cycle’s carboxylation steps has been previously found in MAGs also belonging to the *Thermoplasmatota* from a hypersaline lake in the Kenyan Rift Valley; however, no solid conclusion could be made due to the predicted ACL possessing low similarity (32%) to known ACL sequences (26).

In contrast, MAGs 3300020171_15 and 3300027917_99 encoded both carboxylases required for the rTCA1 cycle as well as both subunits of the ACL (ACLa and ACLb), which had sequence similarities of 47% to 56.6% to reference sequence from *C. limicola*. A predicted membrane-embedded quinol: fumarate reductase as well as the predicted capacity for hydrogen-based chemolithotrophy (Fig. 2 and Tables S4 and S6) was also found in both MAGs, thus together providing the so far strongest support for the presence of rTCA1 cycle in the *Thermoplasmatota*.

The rTCA1 cycle was also identified in several MAGs belonging to the phylum *Nitrospirota*. Most of the MAGs belong to the nitrite-oxidizing order *Nitrospirales*, consistent with previous observation (17). However, for the first time, the complete set of enzymes for the rTCA cycle was here identified in organisms of the orders 2–01-FULL-66–17 (class *Nitrospira*) and the *Thermodesulfovibrionales* (MAG ID 3300026280_26), which all lacked the genetic capacity for nitrite-oxidation, but instead appear to rely on the oxidation of hydrogen, sulfite, and/or sulfide for energy generation. Similarly, the rTCA cycle in bacterial order UBA7883 (phylum *Nitrospinota*) also does not appear to rely on the oxidation of nitrite, but instead on hydrogen, sulfide, or sulfite.

We also found genes for the rTCA1 cycle in the *Elusimicrobiota* (4 MAGs with >97% pathway completeness), which is a novel finding for this phylum and appears to be coupled to sulfite and hydrogen oxidation (Fig. 2 and Tables S4 and S6). While members of this phylum have been reported to be facultative autotrophic, this capacity has so far only been attributed to the WLP (27).

### The 3-hydroxypropionate bi-cycle beyond the Chloroflexota

The 3HP bi-cycle was first described in green, nonsulfur bacterium *Chloroflexus aurantiacus* (19, 28) and since then has been commonly and exclusively attributed to the phylum *Chloroflexota* (29, 30). However, enzymes for this pathway (e.g. 2-methylfumaryl-CoA isomerase, malonyl-CoA reductase, and propionyl-CoA synthase) have been recently detected in organisms belonging to the phyla *Actinobacteriota* (formerly *Actinobacteria*) as well as the classes *Alpha-* and *Gammaproteobacteria* within the *Proteobacteria* [recently renamed “*Pseudomonadota* (31)], raising speculation about their ability to undertake autotrophy based on the 3HP bi-cycle (32). Our analysis also detected the key enzymes for this cycle in 27 MAGs of the phylum *Actinobacteriota*, however the average pathway completeness was only 68.6%, which limits the support for the presence of the 3HP bi-cycle (Table S1). Instead, we propose that the detected enzymes are used to assimilate various organic substrates (i.e. glycolate, acetate, propionate, 3-hydroxypropionate, lactate, butyrate, or succinate), similar to what has been described for *C. aurantiacus* (33) (Supplementary Material and Fig. S2).

**Fig. 3.**
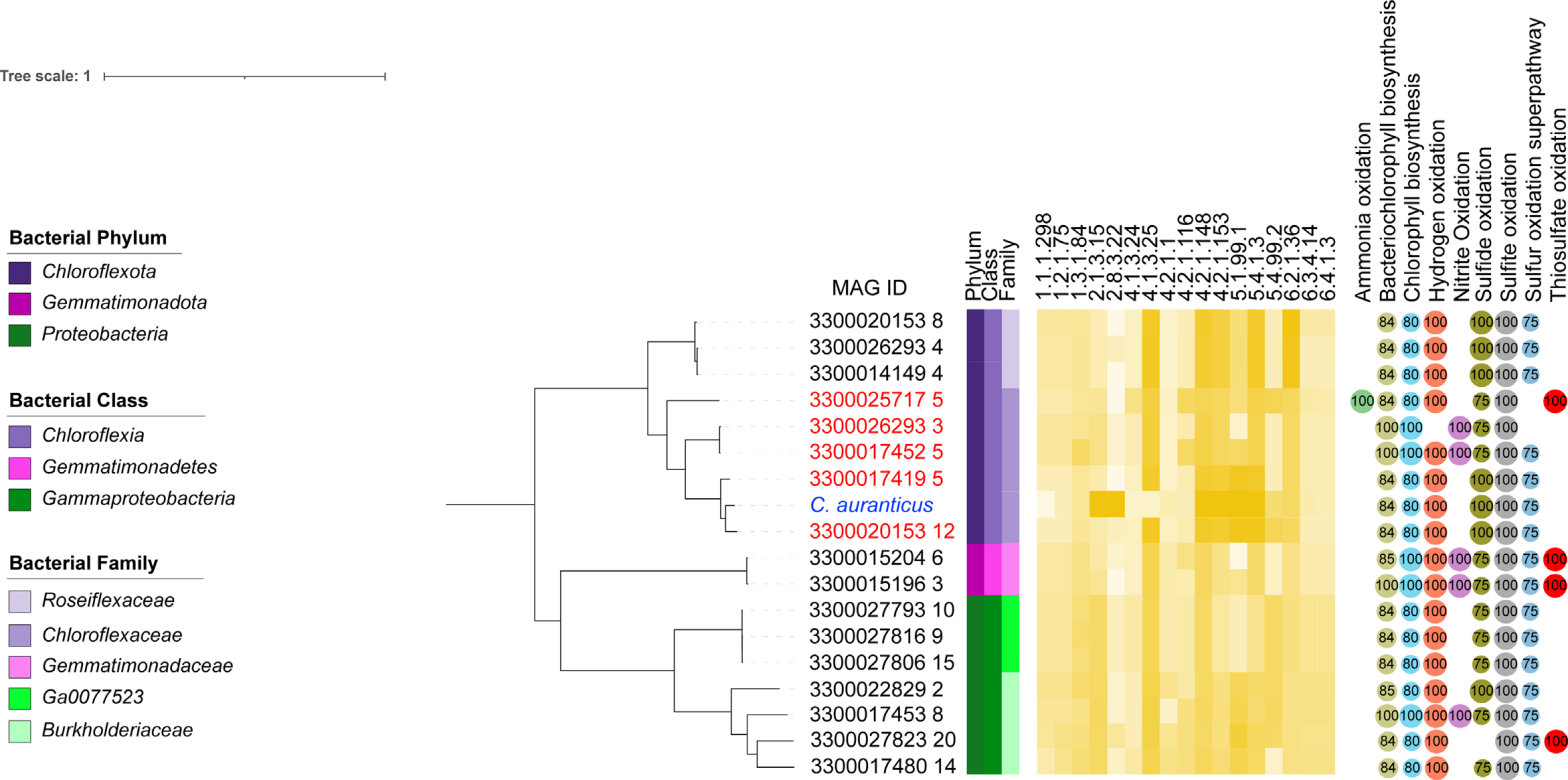
Phylogenetic tree showing the distribution of the 3HP cycle in novel bacterial taxa and in known organisms from the family *Chloroflexaceae* (blue: reference genome; red: MAGs assigned to the same family as the reference genome). Identity of the enzymes involved in the pathway against the UniRef database are shown in yellow heat scale. 1.1.1.298: 3-hydroxypropionate: NADP + oxidoreductase; 1.2.1.75: malonate semialdehyde: NADP + oxidoreductase (malonate semialdehyde-forming); 1.3.1.84: acrylyl-CoA reductase; 2.1.3.15: acetyl-CoA carboxyltransferase; 2.8.3.22: succinyl CoA: L-malate CoA transferase; 4.1.3.24: beta-methylmalyl CoA lyase; 4.1.3.25: S,-citramalyl-CoA lyase; 4.2.1.1: carbonic anhydrase; 4.2.1.116: 3-hydroxypropionyl-CoA dehydratase; 4.2.1.148: beta-methylmalyl-CoA dehydratase; 4.2.1.153: mesaconyl-C4-CoA hydratase; 5.1.99.1: methylmalonyl-CoA epimerase; 5.4.1.3: mesaconyl-C1-CoA-C4-CoA transferase; 5.4.99.2: methylmalonyl-CoA mutase; 6.2.1.36: 3-hydroxypropionate: CoA ligase; 6.3.4.14: biotin carboxylase; 6.4.1.3: propionyl-CoA carboxylase. Numbers in circles refer to completeness of diagnostic pathways for energy generation. Black squares in the heatmap refers to missing enzymes. The tree was rooted with a MAG from the *Actinobacteriota*, which is not shown here.

As for the *Proteobacteria*, we discovered MAGs with adjusted pathway completeness above the required threshold within the families *Ga0077523* and *Burkholderiaceae* (both class *Gammaproteobacteria*, order *Burkholderiales*), both of which have also the predicted capacity of photosynthesis and oxidation of various inorganic electron donors (Fig.   3).

The three MAGs of the family *Ga0077523* belong to the same genus (*Ga0077523*) and contain all genes for the glyoxylate salvage in the same operon, a characteristic conserved among organisms known to fix carbon through the 3HP bi-cycle (Supplementary Material) (33). Similarly, the four *Burkholderiaceae* MAGs have the genomic potential do fix carbon through the 3HP bi-cycle and display a similar organization of the genes related to the glyoxylate salvage (Supplementary Material).

We also detected all genes required for the 3HP bi-cycle for the first time in the phylum *Gemmatimonadota* (two MAGs assigned to the order *Gemmatimonadales*), whose members are usually found in plant rhizospheres where they thrive in carbon- and nitrogen-depleted environments (34, 35). *Gemmatimonadota* have also recently been found in hadal trenches, where they are considered to play an important role in organic carbon turnover (36). The arrangement of genes for the glyoxylate salvage pathway were also similar to those the other MAGs or reference organism. (Supplementary Material and Fig. S3). The predicted capacity for autotrophy in these *Gemmatimonadales* might be linked to the oxidation of various inorganic electron donors (Fig. 3 and Tables S4 and S6).

### An expanded range of the CBB cycle

The CBB cycle is present in all eukaryotic plants and cyanobacteria and widespread in several chemoautotrophic bacteria and purple phototrophic bacteria (37–39). Consistent with the previous literature, we detected the genetic potential for the CBB in members of the phyla *Actinobacteriota, Cyanobacteriota, Chloroflexota, CPR SAR324* (a former clade within the *Deltaproteobacteria*), *Gemmatimonadota, Leptospirae, Proteobacteria, Methylomirabilota* and *Verrucomicrobiota* and specific orders and families within them (40–44) (Supplementary Material and Table S1).

**Fig. 4.**
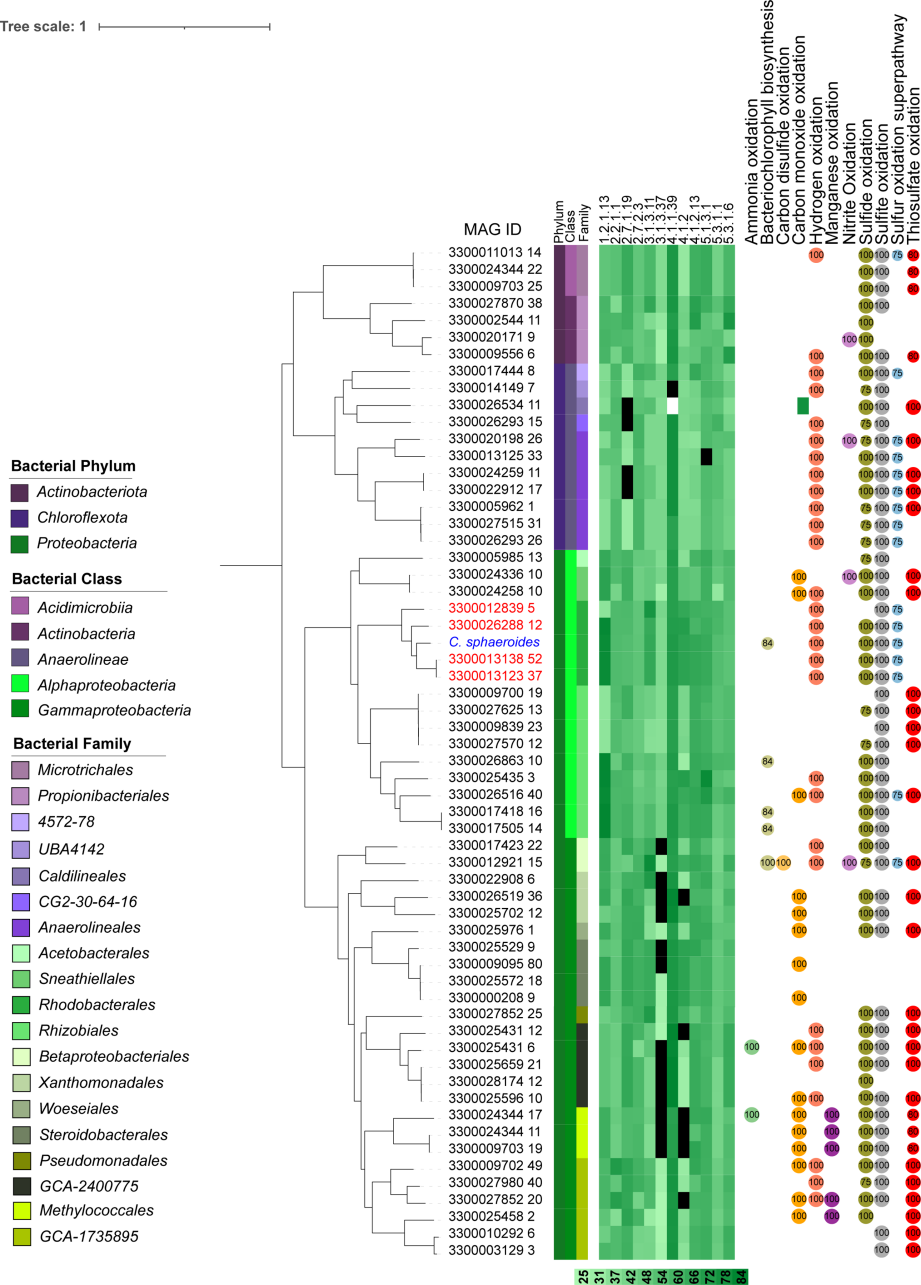
Phylogenetic tree showing the distribution of the CBB cycle in novel bacterial taxa and in known organisms from the family *Rhodobacteraceae* (blue: reference genome; red: MAGs assigned to the same family as the reference genome Identity of the enzymes involved in the pathway against the UniRef database are shown in green heat scale). 1.2.1.13: glyceraldehyde-3-phosphate dehydrogenase; 2.2.1.1: D-fructose 6-phosphate: D-glyceraldehyde-3-phosphate glycolaldehydetransferase; 2.7.1.19: phosphoribulokinase; 2.7.2.3: phosphoglycerate kinase; 3.1.3.11: fructose 1,6-bisphosphatase; 3.1.3.37: sedoheptulose bisphosphatase; 4.1.1.39: ribulose bisphosphate carboxylase; 4.1.2: sedoheptulose-1,7-bisphosphate aldolase, 4.1.2.13: fructose-bisphosphate aldolase; 5.1.3.1: pentose-5-phosphate 3-epimerase; 5.3.1.1: triosephosphate isomerase; 5.3.1.6: ribose 5-phosphate isomerase. Numbers in circles refer to completeness of diagnostic pathways for energy generation. Black squares in the heatmap refers to missing enzymes. The tree was rooted with a MAG from the *Gemmatimonadota*, which is not shown here.

Despite its widespread and extensively studied distribution, we found genomic evidence for the CBB cycle in many new taxa and these includes members of the order *Microtrichales* (phylum *Actinobacteriota*, class *Acidimicrobiia*) and Propionibacteriales (phylum *Actinobacteriota*, class *Actinobacteria*) (Fig. 4). These MAGs have the genetic potential to perform the sedoheptulose-1,7-bisphosphate (SBPase) variant of the cycle, where erythrose 4-phosphate and dihydroxyacetone phosphate are condensed into sedoheptulose 1,7-biphosphate by an aldolase (45). We also found here first evidence for its presence in members of the families *Beijerinckiaceae, Methyloligellaceae, Pseudoxanthobacteraceae*, and *UBA4765*, which all belong to the order *Rhizobiales* that has recently been shown to contain autotrophic organisms (46).

Novel is also the finding of a complete set of CBB genes in the family *Acetobacteraceae* (order *Acetobacterales*, class *Alphaproteobacteria*). It has been previously reported that organisms in this family encode most of the enzymes required for the CBB, but no genetic evidence for a phosporibulokinase (PRK) was found (47). In contrast, we detected in MAG 3300005985_13 of this family a protein sequence for PRK I with a sequence similarity of 69.4% to the reference PRK I from *Cereibacter sphaeroides*. For the first time, we were also able to identify the genes for a complete CBB cycle in the order *Sneathiellales*, which have so far only been described as chemoheterotrophic (48). Both MAGs (3300024336_10 and 3300024258_10) identified are unassigned at the family and genus level, which limits further comparison with the literature, but they have the predicted capacity for chemolithotrophy via the oxidation of carbon monoxide, hydrogen, nitrite, sulfide, sulfite and/or thiosulfate.

A full set of genes for the CBB cycle was also found in two MAGs of the family *Marinicellaceae* and one MAG assigned to the family *Rhodanobacteraceae* (both order *Xanthomonadales*, class *Gammaproteobacteria*). *Xanthomonadales* are primarily chemoorganoheterotrophs, although evidence for chemolithotrophy based on sulfur oxidation has been found (49). We were able to detect genes encoding for the oxidation of carbon monoxide, sulfide, sulfite, and/or thiosulfate in the *Marinicellaceae* MAGs (3300025702_12 and 3300026519_36), while for the *Rhodanobacteraceae* MAG (3300022908_6), no pathways for lithotrophy or photosynthesis could be found.

The order GCA-1,735,895 (class *Gammaproteobacteria*) has been previously inferred to be mixotrophic, but no CFPs were identified (50). Our findings indicate that its autotrophic growth is based on the CBB cycle as well as the potential to generate energy through the oxidation of carbon monoxide, hydrogen, manganese, nitrite, sulfide, sulfite, or thiosulfate (Fig. 4 show six representative MAGs and for the remainder see Tables S1, S4, and S6). Similarly, organisms assigned to the order GCA-2,400,775 have been predicted to be chemolithotrophic, even though no CFP had been proposed (51). In our analysis, 96 MAGs assigned to this taxon have genes for the CBB cycle and have the predicted capacity to oxidise ammonia, carbon monoxide, hydrogen, sulfide, sulfite, or thiosulfate (Fig. 4 show five representative MAGs and for the remainder see Tables S1, S4, and S6). We also found all genes for the CBB cycle in organisms assigned to the family *IMCC2047* (order *Pseudomonadales*), which has genes for the oxidation of sulfide, sulfite, and thiosulfate. Within the order *Betaproteobacteriales*, we also detected genomic evidence for the CBB cycle in the family *Ga0077523* (MAG 3300012921_15). This MAGs is predicted to be capable of oxidizing carbon disulfide, hydrogen, nitrite, sulfide, sulfite, sulfur, or thiosulfate, or synthetize bacteriochlorophyll.

Intriguingly, we also found all genes for the CBB cycle with the exception of sedoheptulose bisphosphatase (SBPase) in MAG 3300017423_22 from the order *Betaproteobacteriales* (family *Methylophilaceae*). However, fructose bisphosphatase has been reported to be a bifunctional enzyme that can act as SBPase in some organisms (45, 52). This family is known to be strictly methylotrophic and assimilates methylated compounds through the ribulose monophosphate cycle (RuMP), which shares its reduction steps with the CBB cycle (53), but does not utilize PRK and ribulose 1,5-bisphosphate carboxylase/oxygenase (RuBisCO) (45). This MAGs also lacks genes for methanol oxidation, but instead genes encoding for enzyme to oxidize hydrogen, sulfite, and sulfide. In addition, we detected in this MAG genes encoding for hexulose-6-phosphate synthase (HPS), hexulose-6-phosphate isomerase (PHI), methylene-tetrahydromethanopterin dehydrogenase, and methylene-H4MPT cyclohydrolase, which are part of the recently described reductive hexulose-phosphate (RHP) pathway (53). This pathway is different from the CBB in that PHI isomerizes fructose 6-phosphate to hexulose 6-phosphate, which is then cleaved by HPS into formaldehyde and ribulose 5-phosphate. This pathway has been proposed for an archaeon (53) and our genomic evidence suggest that it might also be operational in the bacterial family *Methylophilaceae*. This proposition for autotrophy is also supported by the fact that the majority of the genes for the RHP pathway are collocated in the same genomic region of MAG 3300017423_22 (Fig. S4).

Growth supported by the CBB cycle has been previously described for members of the methanotrophic order *Methylococcales*, but only for the family *Methylococcaceae* (54). We found all genes for the CBB in three MAGs assigned to the family *Methylomonadaceae* of that order. This family was thought to be strictly heterotrophic (55), but our results rather support the notion that they can also grow autotrophically, possibly generating energy from the oxidation of ammonia, carbon monoxide, manganese, sulfide, sulfite, or thiosulfate. Growth using the CBB cycle has also been predicted for members of the family *Woeseiaceae* of the order *Steroidobacterales* (56). Here, we expand the phylogenetic breadth of the CBB within this order by discovering also all necessary genes for the CBB cycle in MAGs of the family *Steroidobacteraceae* and we show that MAGs 3300009095_80 and 3300000208_9 have the potential to oxidize carbon monoxide.

Recently, a new form I’ of RuBisCO was described in the phylum *Chloroflexota*, which shares a high similarity with RuBisCO form I, but does not depend on the small subunit to be active (57). Form I’ was found in MAGs belonging to the order *Anaerolineales* (class *Anaerolineae*, phylum *Chloroflexota*) alongside with several other enzymes of the CBB cycle, such as PRK, glyceraldehyde-3-phosphate dehydrogenase (GAPD), transaldolase (TAL), and FBPase, suggesting autotrophic growth. Our results suggest that this mechanism might also span in the *Caldilineales* (class *Anaerolineae*), as one MAGs in that order have genes for PRK, GDAP, FBPase, and TAL and also only encode for the large subunit of RuBisCO, which has 78.87% sequence similarity to the form I’ reference sequence (PDB: 6URA_A). Similar results were found for MAGs in the placeholder orders *CG2-30-64–16, 4572–78*, and *UBA4142* of the *Anaerolineae*, suggesting that RuBisCO form I’ might be a synapomorphy for the class, not only for the order.

### The expanded distribution of the dicarboxylate/4-hydroxybutyrate cycle in the Archaea

**Fig. 5.**
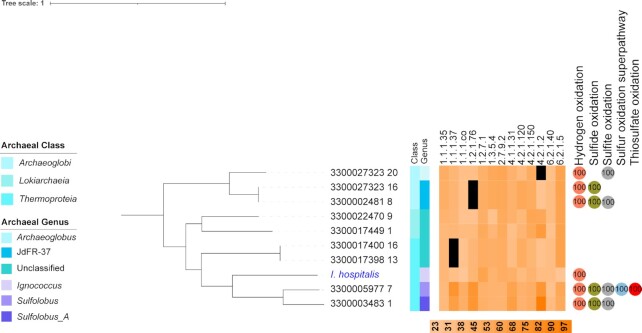
Phylogenetic tree showing the distribution of the DC/HB cycle in novel archaeal taxa and the reference genome of *I. hospitalis*. Identity of the enzymes involved in the pathway against the UniRef database are shown in orange heat scale. 1.1.1.35: S-3-hydroxybutyryl-CoA dehydrogenase; 1.1.1.37: malate dehydrogenase; 1.1.1.co: succinate semialdehyde reductase; 1.2.1.76: succinyl-CoA reductase; 1.2.7.1: pyruvate synthase (ferredoxin-dependent); 1.3.5.4: fumarate reductase; 2.7.9.2: phosphoenolpyruvate synthetase; 4.1.1.31: phosphoenolpyruvate carboxylase; 4.2.1.120: 4-hydroxybutyryl-CoA dehydratase; 4.2.1.150: S,-3-hydroxybutanoyl-CoA dehydrogenase; 4.2.1.2: fumarase; 6.2.1.40: 4-hydroxybutyryl-CoA synthetase; 6.2.1.5: succinyl-CoA synthetase. Numbers in circles refer to completeness of diagnostic pathways for energy generation. Black squares in the heatmap refers to missing enzymes. The tree was rooted with a MAG from the *Methanobacteriota*, which is not shown here.

The dicarboxylate/4-hydroxybutyrate (DC/HB) cycle was initially described in *Ignicoccus hospitalis*, an hydrogen-oxidizing and sulfur-reducing archaeon (58). The DC/HB cycle’s conversion of succinyl-CoA to acetyl-CoA uses the same enzymes as the HP/HB cycles, but the regeneration of the former involves the carboxylation of acetyl-CoA into pyruvate catalyzed by an oxygen-sensitive, ferredoxin-dependent pyruvate synthase. This cycle appeared to be restricted to anaerobic archaea of the orders *Sulfolobales* and *Thermoproteales* (phylum *Thermoproteota*, class *Thermoproteia*) (59, 60), but our analysis substantially expands its range within the archaeal domain (Fig. 5 and Table S1).

Two MAGs assigned to the class *Lokiarchaeia* (phylum *Asgardarchaeota*) contained all genes for the DC/HB cycle. Member of the *Asgardarchaeota* have been suggested to be capable of growing autotrophically, through the WLP or even through reactions mediated by a type III-like Rubisco (Bulzu, P et. al, 2019) (61, 62), but also, as we show here, possibly through the DC/HB cycle. Further work is required to determine which CFPs are used by this metabolically diverse class and phylum.

**Fig. 6.**
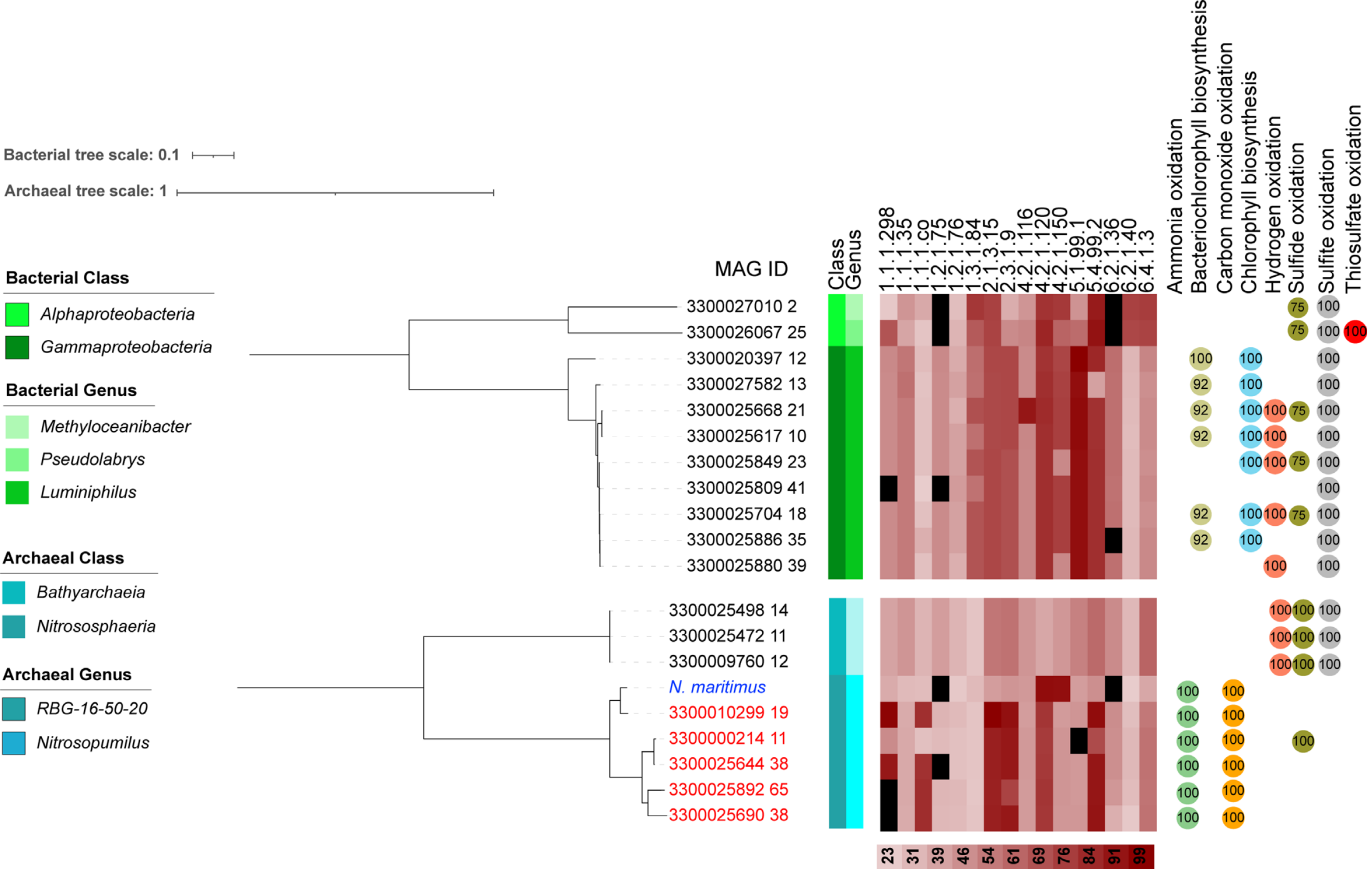
Phylogenetic tree showing the distribution of the HP/HB cycle in novel bacterial and archaeal taxa and in known organisms from the family *Nitrosopumilaceae* (blue: reference genome; red: MAGs assigned to the same family as the reference genome). Identity of the enzymes involved in the pathway against the UniRef database are shown in red heat scale. 1.1.1.298: malonate semialdehyde reductase; 1.1.1.35: S,-3-hydroxybutyryl-CoA dehydrogenase; 1.1.1.co: succinate semialdehyde reductase; 1.2.1.75: malonyl-coenzyme A reductase; 1.2.1.76: succinyl-CoA reductase; 1.3.1.84: acrylyl-CoA reductase; 2.1.3.15: acetyl-CoA carboxytransferase; 2.3.1.9: acetoacetyl-CoA beta-ketothiolase; 4.2.1.116: 3-hydroxypropionyl-CoA dehydratase; 4.2.1.120: 4-hydroxybutyryl-CoA dehydratase; 4.2.1.150: S,-3-hydroxybutanoyl-CoA dehydrogenase; 5.1.99.1: methylmalonyl-CoA epimerase; 5.4.99.2: methylmalonyl-CoA mutase; 6.2.1.36: 3-hydroxypropionyl-CoA synthetase; 6.2.1.40: 4-hydroxybutyryl-CoA synthetase; 6.4.1.3: propionyl CoA carboxylase. Numbers in circles refer to completeness of diagnostic pathways for energy generation. Black squares in the heatmap refers to missing enzymes.

And finally, we find, for the first time, genomic evidence for the DC/HB cycle in the genus *Sulfolobus* (Fig. 5), whose members have been reported to be microaerophilic thermophiles that can oxidise reduced sulfur compounds (Fig. 6) (63). Such growth conditions might allow oxygen-sensitive enzymes of the DC/HB cyle, such as the ferredoxin-dependent pyruvate synthase, to be active (22). To the best of our knowledge, *Sulfolobus* has so far been thought to grow autotrophically exclusively through the HP/HB cycle (60, 21, 64). Our genomic evidence suggests that it can also grow through the DC/HB cycle, because of the presence of all the enzymes required for the regeneration of acetyl-CoA from succinyl-CoA (Fig. 5).

### The 3-hydroxypropionate/4-hydroxybutyrate cycle in the Archaea and Bacteria

The HP/HB cycle was first discovered in the thermoacidophilic, iron-oxidizing archaeon *Metallosphaera sedula* (65) and has been more recently described in detail for the ammonium-oxidizing archaeaon *Nitrosopumilus maritimus* (66, 67). So far, this cycle has only been reported for the *Archaea*. The 4-hydroxybutyrate dehydratase (HBD) is commonly considered a key enzyme (65) as it appears in the *Archaea* to be only involved in the HP/HB cycle. In the *Bacteria*, however, HBD is part of different metabolic pathways (i.e succinate fermentation and 4-aminobutanoate degradation) (68).

As expected, we found ample of genomic evidence for this cycle in MAGs belonging to the archaeal phylum *Thermoproteota* as well as the classes *Nitrososphaeria* (order *Nitrososphaerales*) and *Thermoprotei* (order *Sulfolobales*) (Table S1). We also found evidence for this cycle in the genus *Archaeoglobus* (phylum *Halobacteriota*, order *Archaeoglobales*) (Table S1). However, it has been previously experimentally shown that *Archaeoglobus lithotrophicus* fixes carbon solely through the WLP, even though predicted homologs for HBD were found in its genome (69). It is therefore unclear what role the HP/HB cycle enzymes have in this genus.

Novel here is the discovery of the HP/HB cycle in three MAGs of the family RBG-16–50–20 within the class *Bathyarchaeia*. These MAGs have the genomic potential to encode all the enzymes required for carbon fixation through the 3-hydroxypropionate/4-hydroxybutyrate cycle and this process seems to be supported by oxidation of hydrogen, sulfide, and/or sulfite.

Unexpectedly, we found genomic evidence of the HP/HB cycle in the *Bacteria*. Eight MAGs belonging to the genus *Luminiphilus* (phylum *Proteobacteria*, class *Gammaproteobacteria*, order *Pseudomonadales*, family *Halieaceae*) encoded all the enzymes for this cycle (Fig. 6 and Table S1). We have also detected genes for the synthesis of (bacterio-) chlorophyll in most of these MAGs. Members of the genus *Luminiphilus* have previously been described to use organic carbon sources and have a full photosynthetic apparatus but lack PRK or RuBisCO genes. They were therefore thought to be photoheterotrophic organisms (70), however, our observation shows that they are instead likely capable of autotrophic growth through the HP/HB cycle, linking this cycle for the first time also to photosynthesis.

Our observation represents only the second time that the HP/HB cycle has been proposed to exist in the bacterial domain of life, with previous work suggesting its presence in the candidate phylum *SBR1093* and in some organisms within the *Bradyrhizobiaceae* of the order *Rhizobiales* (71). Our results showed that the MAGs 3300026067_25 (genus *Pseudolabrys*) and 3300027010_2 (genus *Methyloceanibacter*) within this order have the potential to fix carbon through this cycle (88% completeness). Further inspection showed this to be due to missing genes for the malonyl-CoA reductase and the 3-hydroxypropionyl-CoA synthetase. Distant homologs, however, may exist for members of both families as manual analysis revealed proteins sharing 30.5% identity across their full lengths with a predicted malonyl-CoA reductase (UniRef cluster UniRef50_A0A0P8CE70) and 28.5% identity with a predicted 3-hydroxypropionyl-CoA synthetase (UniRef cluster UniRef50_A5UY60) for the *Pseudolabrys* MAG. For the MAGs classified as *Methyloceanibacter*, we also found protein sequences sharing 30.1% identity across their entire length with predicted malonyl-CoA reductase and 28.5% identity with 92.2% coverage with a predicted 3-hydroxypropionyl-CoA synthetase sequence of corresponding UniRef clusters. Altogether, this suggests the potential for the HP/HB cycle in these taxa, which also have the predicted capacity of sulfur-based chemolithotrophy (Fig. 6).

Together, these results show that the HP/HB cycle occurs not only in the archaeal domain, but also within the Bacteria. Furthermore, the data support the notion that this cycle is not only restricted to chemolithotrophic organisms, but can also be found in phototrophs.

## Conclusion

Our analysis has revealed that CFPs are much more widespread in the archaeal and bacterial domains of life than previously anticipated. Evolutionarily, this is likely underpinned by complex patterns of horizontal gene transfer and gene loss (7, 43). Future work could monitor the CFP expression for these new taxa found here to better understand their role in global primary production and define how they contribute to microbial carbon sinks in various ecosystems.

## Material and methods

The nucleotide and predicted protein sequences of 52,515 MAGs were obtained from the GEM catalogue, available at JGI Genome Portal with the identifier DOI: 10.1038/s41587-020-0718-6 (12). These MAGs were generated from 3,170 metagenomic samples obtained from a wide range of habitats (including freshwater, marine water, soil, and thermal springs; see Fig. S1 and Table S1).

For each CFP, we defined key enzymes, which are unique to the specific pathway, and built hidden Markov models (HMMs) using the HMMER suite version 3.3 (Table 1). For the models, we only selected sequences that were experimentally proven to catalyze the specific reaction in order to prevent issues related to “genome annotation rot” (72). When only one experimentally validated sequence was available, we did BLASTP searches to select enzymes with an identity higher than 90% from different organisms. HMMsearch results are available in Table S3. For the CBB, we decided to use phosphoribulokinase (PRK) as the key enzyme for our initial searches instead of RuBisCO as the latter has five different isoforms, six different clades, and different subunits for isoforms, which would have complicated HMM building (57). Accession numbers and seed alignments can be found in Table S2. An HMM for the mesaconyl-CoA isomerase (TIGR04253) was previously available (http://tigrfams.jcvi.org/cgi-bin/index.cgi) (73).

All MAGs with a positive hit (*E*-value < 10^−99^) for the key enzymes had the completeness of their CFPs analyzed using GapSeq version 1.1 with the UniProt database version UniRef 2021_01 (74). UniRef stores protein sequence clusters at different resolutions that account for sequence similarities within each cluster, which allow to infer homology between proteins. GapSeq combines searches against protein reference sequence databases, such as UniProt, with pathway and reaction databases (ModelSEED, KEGG, and MetaCyc) in order to infer the presence and completeness of metabolic pathway in a genome. Carboxylation reactions were also considered key reactions for all GapSeq analyses. GapSeq parameters were set to use unreviewed protein sequences only when reviewed sequences were not available for a particular reaction or enzyme function, and a bit score higher than 100 was required (Table S1).

Thresholds for pathway completeness were defined using the complete genomes of well-studied model organisms for each particular CFP (Table 1). Pathway completeness for the model organisms was analyzed by GapSeq. The expected completeness thresholds were set 10% lower than the reference genome values obtained by GapSeq in a conservative measure to avoid false negatives. The observed CFP completeness for the MAGs was then divided by the genome completeness, which was estimated using the CheckM algorithm (75) with lineage-specific marker genes, to obtain the adjusted, observed completeness. A particular CFP was considered to be present if a MAG is adjusted, observed CFP completeness was above the expected completeness threshold. MAGs with estimated genome completeness lower than 75% were generally not considered. MAGs 3300013125_33 and 3300017423_22 were the exception as we manually checked them for the form I RuBisCO.

In most cases, multiple, phylogenetically closely related MAGs with the same predicted CFP were found and this gave confidence that the pathway’s presence in a specific taxon or clade is not due to contaminations. However, in some cases, only one MAG with a particular CFP was recovered for a specific taxon. In those cases, genomic regions flanking the CFP genes were checked for potential mis-assemblies using GC content and taxonomic assignment of neighbouring genes.

Taxonomy was assigned to MAGs using the GTDB version 95 and GTDB-Tk toolkit version 94 (76). Mapping of taxonomic names in GTDB to those used in the taxonomy of the National Center for Biotechnology Information (NCBI) can be found at http://gtdb.ecogenomic.org. Unrooted bacterial and archaeal phylogenetic trees based on 120 phylogenetic marker genes for bacteria and 122 marker genes for archaea were generated by GTDB-Tk and visualized using iTOL (77).

Energy-generating pathways were inferred using GapSeq outputs focusing on 34 diagnostic pathways in the MetaCyc database, which are involved in the oxidation of 11 different inorganic compounds. In addition, we determined the potential for photosynthesis through the MetaCyc superpathways for chlorophyll and bacteriochlorophyll syntheses. Completeness of the pathways was determined without considering MAG completeness and values of >75% are reported for the most complete diagnostic pathways for each compound. The percentage shown in the dot plot refers to the most complete oxidation pathway for a specific compound (e.g. if four different sulfide oxidation pathways were available to be analyzed, then the percentage in the plot represents the percentage of the most complete pathway). A list of the analyzed pathways and their pathway completeness as well as all genes found are available in Tables S4 and S6.

## Supplementary Material

pgac226_Supplemental_Files

## Data Availability

Download for the 52,515 MAGs and their amino acids files is available at https://genome.jgi.doe.gov/GEMs and https://portal.nersc.gov/GEM. HMM models used are available at https://github.com/alegarritano/HMM_CFP.
