# Supplementary material for: Carbon fixation pathways across the bacterial and archaeal tree of life
Source: PNAS Nexus. 2022 Oct 4;1(5):pgac226. doi: 10.1093/pnasnexus/pgac226 (PMC9802188; doi:10.1093/pnasnexus/pgac226)
Supplement: pgac226_Supplemental_Files [file pgac226_supplemental_files.zip › PNASNEXUS-PNASNEXUS-2022-00227-T-s01.docx]

**Supplementary Information for**

Carbon fixation pathways across the bacterial and archaeal tree of life

Authors: Alessandro N. Garritano^1^, Weizhi Song^1^, Torsten Thomas^1*^

1 – Centre for Marine Science and Innovation, School of Biological, Earth and Environmental Sciences, Faculty of Science, The University of New South Wales, Kensington, NSW 2052, Australia

*Corresponding author

Prof. Torsten Thomas Centre for Marine Science and Innovation School of Biological, Earth and Environmental Sciences The University of New South Wales, NSW 2052, Australia Ph + 61 (0) 2 938 53467 Email: [t.thomas@unsw.edu.au](mailto:t.thomas@unsw.edu.au)

**This PDF file includes:**

Supplementary Information

Supplementary Figures 1-4

**Supplementary Results and Discussion**

***Assimilation of organic compounds using 3HP bi-cycle enzymes in the Actinobacteriota***

The 3HP bi-cycle is most complex CFP known so far. It requires 13 enzymes that catalyze 19 reactions. However, several organisms make use of incomplete versions of this cycle to obtain energy from organic molecules available in the environment, such as acetate, lactate, succinate, butyrate, 3-hydroxypropionate and glycolate (Figure S2) (1). For instance, all the MAGs assigned to the family UBA11606 (order *Microtricales*, class *Acidimicrobiia*) with enzymes of the 3HP bi-cycle lack the genes for methylmalonyl-CoA mutase and methylmalyl-CoA dehydratase. However, they still have genes encoding for several other enzymes of the pathway. Thus, there is genetic potential to assimilate fermentation products, such as propionate. Aromatic compounds could also be metabolized into succinyl-CoA and converted into acetyl-CoA through this pathway (2). Co-assimilation examples in other microorganisms are found with the assimilation of glycolate released by cyanobacteria or algae during photorespiration and its further conversion into acetyl-CoA or the metabolism of ß-alanine or DMSP into succinyl-CoA (3).

***Genomic arrangement of genes for the glyoxylate salvage pathway in Proteobacteria with the 3HP bi-cycle***

The genes for the glyoxylate salvage pathway were found in the same order as an apparent operon structure in the MAGs classified as *Burkholderiaceae* and Gemmatimonadales as well as the model organisms *Roseiflexus* sp. and *Chloroflexus* sp. (Figure S2). The genes for SmtAB, Mct, Mcl, Mch were found in tandem in the MAGs belonging to family Ga0077523, however the *meh* gene was consistently found in a different genomic region. In order to investigate the possibility for contamination, the flanking regions of the operons were analyzed and no contamination in these genomic regions was detected.

***Specific taxa previously known to have the CBB***

As reported in the literature, the CBB is found widespread across several different phyla. Specific lower taxa with genetic evidence for the CBB cycle comprise of MAGs assigned to the orders Rhizobiales, Geminicoccales, Azospirilalles, Sphingomonadales, Rhodobacterales, and Rhodospirillales, (class Alphaproteobacteria) and Mariprofundales (class Zetaproteobacteria), consistent with previous literature (4–10). Other examples include MAGs within the orders Acidiferrobacterales, Beggiatoales, Betaproteobacteriales, Chromatiales, Ectothiorhodospirales, Halothiobacillales, Nitrosococcales, Thiohalobacterales (former Chromatiales), Thiohalomonadales (former Chromatiales), Thiohalospirales (former Chromatiales), Thiomicrospirales, Thiotrichales, Methylococcales, Neviskiales (former Salinisphaerales), Nitrococcales (former Chromatiales), Steroidobacterales, UBA10353 (former Acidiferrobacterales) and Woeseiales (former Chromatiales) assigned to the Gammaproteobacteria class (9, 11–16). Specific families, for which we found evidence for CBB-based autotrophy in both our MAGs and the literature, include also member of the *Burkholderiaceae*, *Ferrovaceae*, *Gallionellaceae*, *Hydrogenophilaceae*, *Nitrosomonadaceae*, *Rhodocyclaceae* and *Sulfuricellaceae*. (17–23).

**Supplementary Figures:**

**
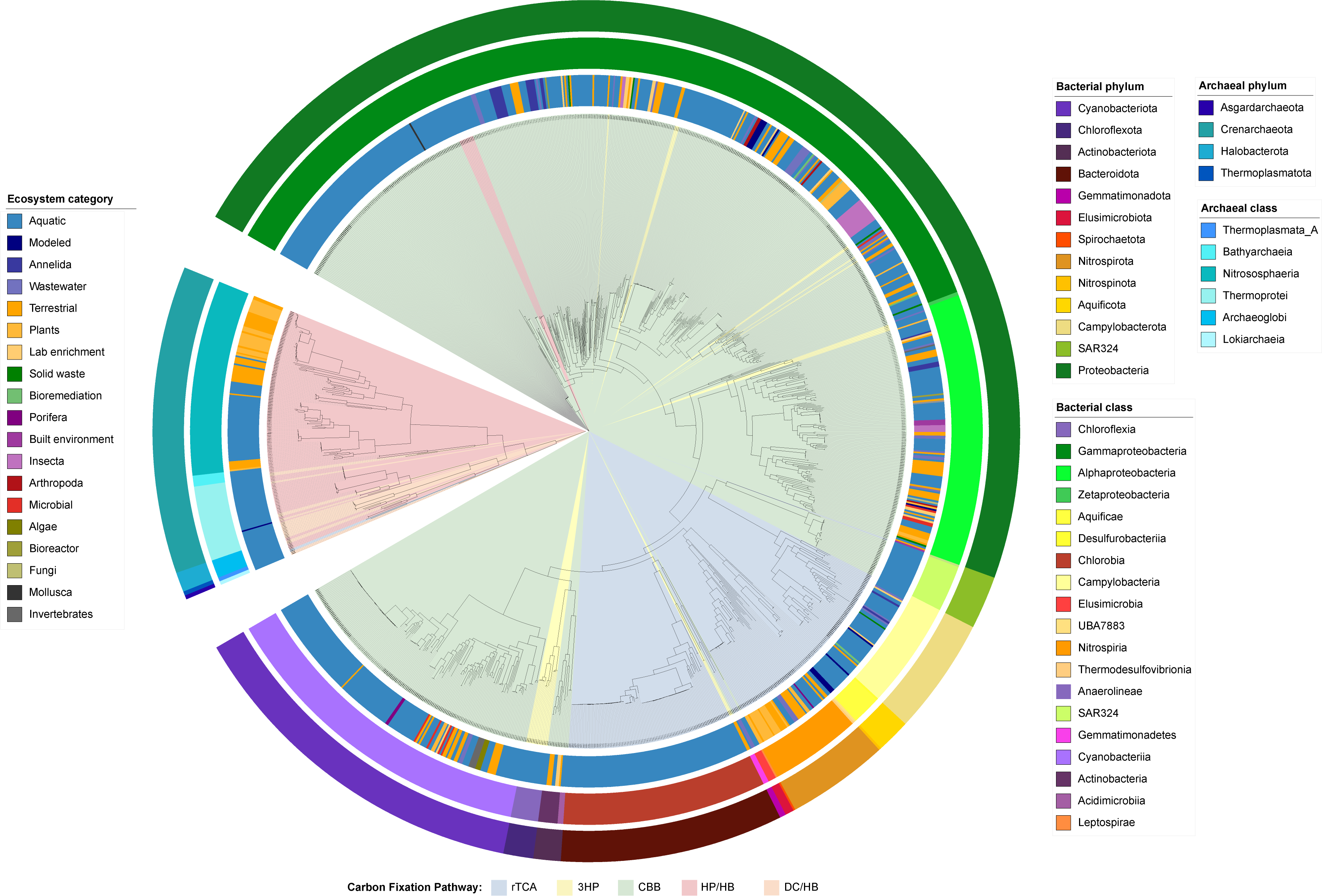
**

**Figure S1:** Phylogenetic tree representing the distribution of the 1005 MAGs across the habitat in which they were sampled. The inner ring represents the ecosystem category of the MAGs, the middle ring represents their classes and the outer ring, their phylum. Shading in the cycle show the five different CFPs analyzed.


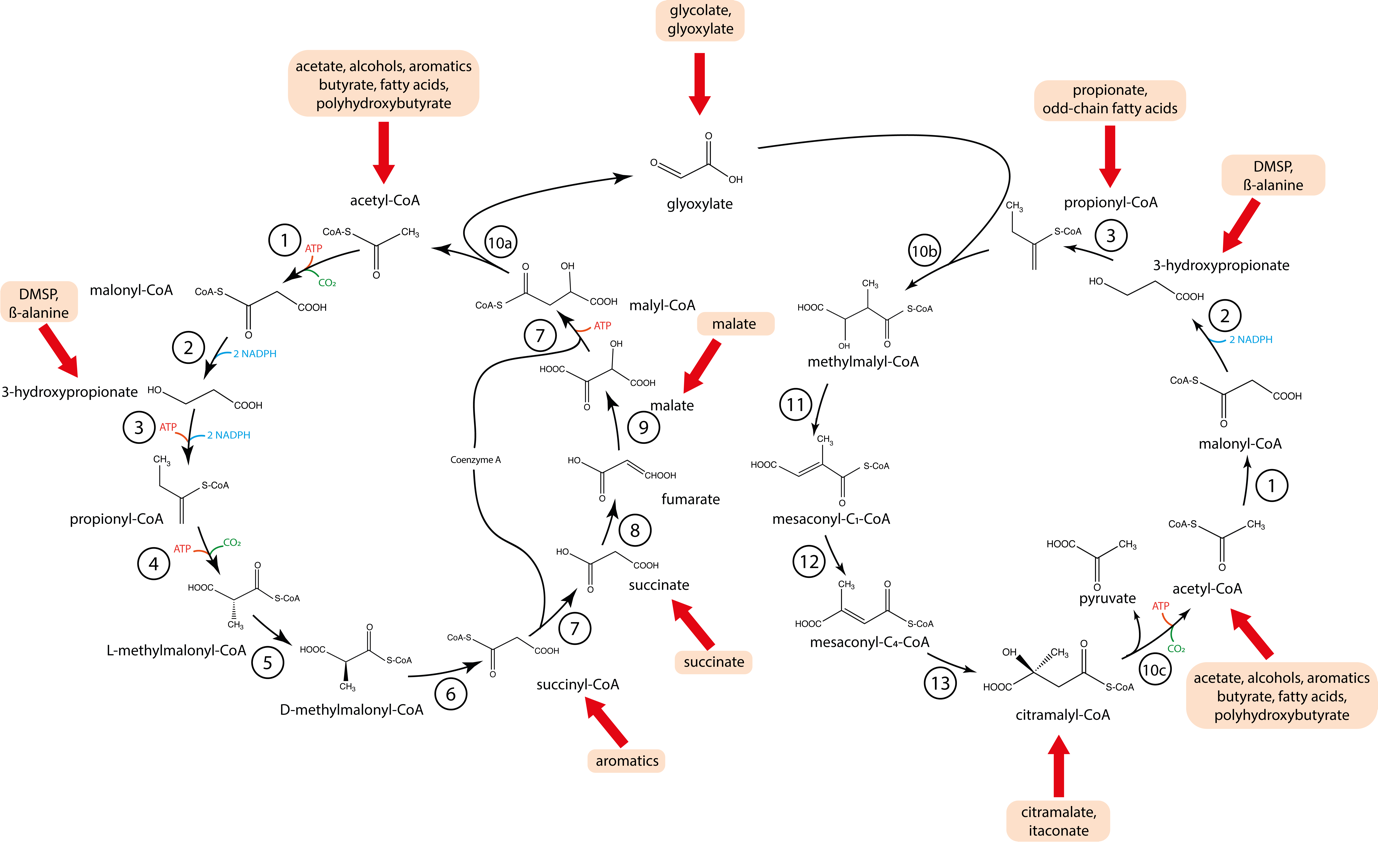


Figure S2: Assimilation of organic compounds using 3HP bi-cycle enzyme. Potential entry points for various organic substrates are shown: 1, acetyl-CoA carboxylase; 2, malonyl-CoA reductase; 3, propionyl-CoA synthase; 4, propionyl-CoA carboxylase; 5, methylmalonyl-CoA epimerase; 6, methylmalonyl-CoA mutase; 7, succinyl-CoA:malate-CoA transferase; 8, succinate dehydrogenase; 9, fumarate hydratase; 10a, malyl-CoA lyase; 10b, methylmalyl-CoA lyase; 10c, citramalyl-CoA lyase; 11, methylmalyl-CoA dehydratase; 12, mesaconyl- CoA C1:C4 CoA transferase; 13, mesaconyl-C4-CoA hydratase.


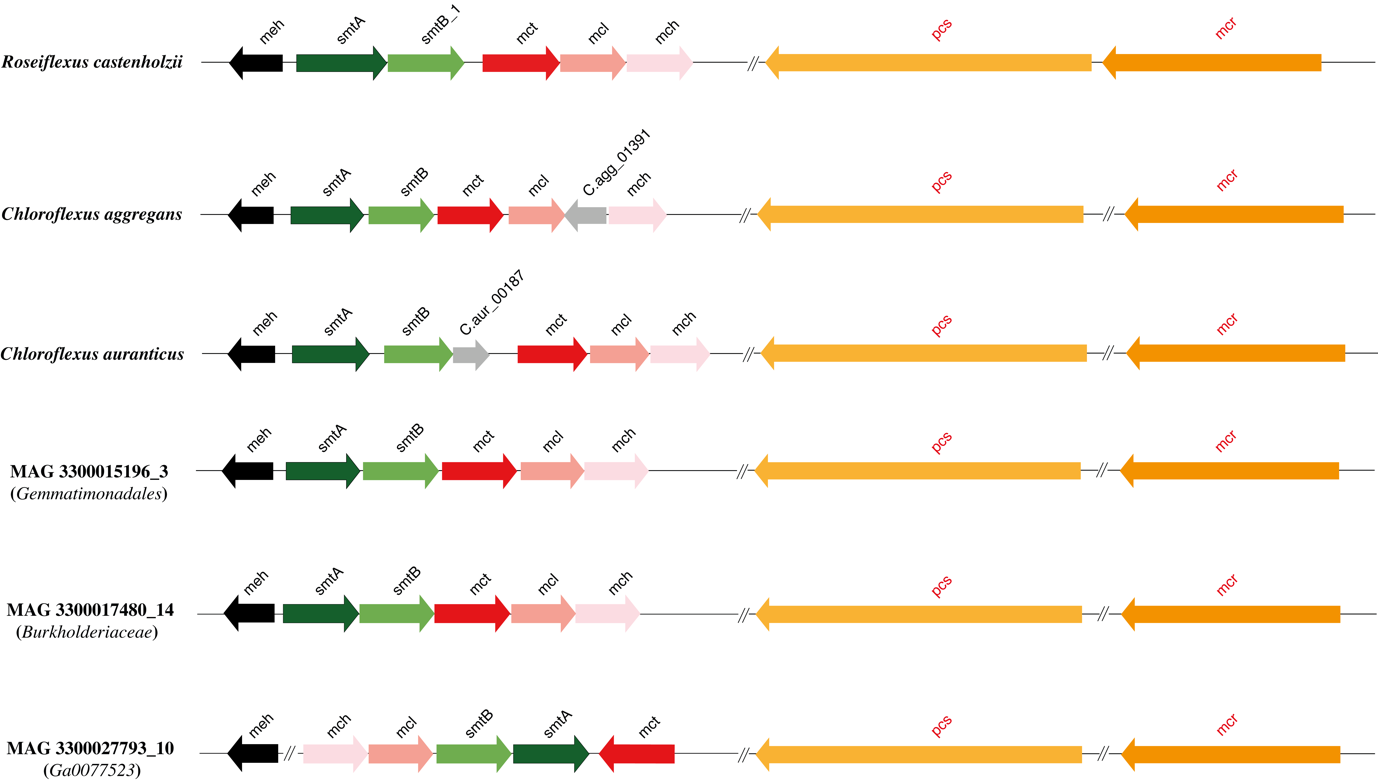


**Figure S3:** Genomic arrangement of genes for the glyoxylate salvage pathway and some of the 3HP bi-cyle. Mesaconyl-C4-CoA hydratase (meh), succinyl-CoA:malate CoA transferase (smtAB), mesaconyl-CoA C1:C4 CoA transferase (mct), (S)-malyl-CoA/methylmalyl CoA/citramalyl-CoA lyase (mcl), mesaconyl-C1-CoA hydratase (mch), propionyl-CoA synthetase (pcs), malonyl-CoA reductase (mcr). Grey arrows show open reading frames (ORFs) of unknown function. Genes in red are associated with the 3HP bi-cyle, while genes in black are related to the glyoxylate salvage.


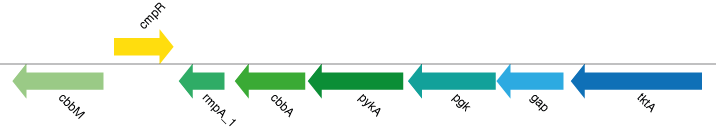


**Figure S4:** Genomic arrangement of gene for the RHP/CBB cyle in MAG 3300017423_22 (order Betaproteobacteriales, family *Methylophilacea*). cbbM, ribulose bisphosphate carboxylase; cmpR, HTH-type transcriptional activator; rmpA_1, 3-hexulose-6-phosphate synthase; cbbA, fructose-bisphosphate aldolase; pykA, pyruvate kinase; pgk, phosphoglycerate kinase; gap, glyceraldehyde-3-phosphate dehydrogenase; tktA, transketolase 1.

**SI References**

1. J. Zarzycki, V. Brecht, M. Müller, G. Fuchs, Identifying the missing steps of the autotrophic 3-hydroxypropionate CO2 fixation cycle in Chloroflexus aurantiacus. *Proc. Natl. Acad. Sci. U. S. A.* **106**, 21317–21322 (2009).

2. P. Ruiz-Fernández, S. Ramírez-Flandes, E. Rodríguez-León, O. Ulloa, Autotrophic carbon fixation pathways along the redox gradient in oxygen-depleted oceanic waters. *Environ. Microbiol. Rep.* **12**, 334–341 (2020).

3. J. Zarzycki, G. Fuchs, Coassimilation of organic substrates via the autotrophic 3-hydroxypropionate bi-cycle in Chloroflexus aurantiacus. *Appl. Environ. Microbiol.* **77**, 6181–6188 (2011).

4. J. K. Zorz, *et al.*, A shared core microbiome in soda lakes separated by large distances. *Nat. Commun.* **10**, 4230 (2019).

5. M. V. Orlova, *et al.*, Genomic insights into metabolic versatility of a lithotrophic sulfur-oxidizing diazotrophic Alphaproteobacterium Azospirillum thiophilum . *FEMS Microbiol. Ecol.* **92**, fiw199 (2016).

6. C. R. Anderson, *et al.*, Aurantimonas manganoxydans, sp. nov. and Aurantimonas litoralis, sp. nov.: Mn(II) oxidizing representatives of a globally distributed clade of alpha-proteobacteria from the order rhizobiales. *Geomicrobiol. J.* **26**, 189–198 (2009).

7. A. C. C. Pires, *et al.*, Comparison of bacterial communities associated with Xestospongia testudinaria, sediment and seawater in a Singaporean coral reef ecosystem. *J. Mar. Biol. Assoc. United Kingdom* **99**, 331–342 (2019).

8. T. Brinkhoff, H. A. Giebel, M. Simon, Diversity, ecology, and genomics of the Roseobacter clade: A short overview. *Arch. Microbiol.* **189**, 531–539 (2008).

9. H. Tong, *et al.*, Microaerophilic Oxidation of Fe(II) Coupled with Simultaneous Carbon Fixation and As(III) Oxidation and Sequestration in Karstic Paddy Soil. *Environ. Sci. Technol.* (2021) https:/doi.org/10.1021/acs.est.0c05791.

10. H. Makita, *et al.*, Mariprofundus micogutta sp. nov., a novel iron-oxidizing zetaproteobacterium isolated from a deep-sea hydrothermal field at the Bayonnaise knoll of the Izu-Ogasawara arc, and a description of Mariprofundales ord. nov. and Zetaproteobacteria classis nov. *Arch. Microbiol.* **199**, 335–346 (2017).

11. S. Lenk, *et al.*, Novel groups of Gammaproteobacteria catalyse sulfur oxidation and carbon fixation in a coastal, intertidal sediment. *Environ. Microbiol.* **13**, 758–774 (2011).

12. K. Umezawa, T. Watanabe, A. Miura, H. Kojima, M. Fukui, The complete genome sequences of sulfur-oxidizing Gammaproteobacteria Sulfurifustis variabilis skN76T and Sulfuricaulis limicola HA5T. *Stand. Genomic Sci.* **11**, 1–8 (2016).

13. N. J. Claassens, *et al.*, Phosphoglycolate salvage in a chemolithoautotroph using the Calvin cycle. *Proc. Natl. Acad. Sci. U. S. A.* **117**, 22452–22461 (2020).

14. N. J. Baxter, *et al.*, The ribulose-1,5-bisphosphate carboxylase/oxygenase gene cluster of Methylococcus capsulatus (Bath). *Arch. Microbiol.* **177**, 279–289 (2002).

15. M. Mußmann, P. Pjevac, K. Krüger, S. Dyksma, Genomic repertoire of the Woeseiaceae/JTB255, cosmopolitan and abundant core members of microbial communities in marine sediments. *ISME J.* **11**, 1276–1281 (2017).

16. M. Crespo-Medina, *et al.*, Salinisphaera hydrothermalis sp. nov., a mesophilic, halotolerant, facultatively autotrophic, thiosulfate-oxidizing gammaproteobacterium from deep-sea hydrothermal vents, and emended description of the genus Salinisphaera. *Int. J. Syst. Evol. Microbiol.* **59**, 1497–1503 (2009).

17. W. F. Fricke, B. Kusian, B. Bowien, The genome organization of Ralstonia eutropha strain H16 and related species of the Burkholderiaceae. *J. Mol. Microbiol. Biotechnol.* **16**, 124–135 (2008).

18. D. B. Johnson, K. B. Hallberg, S. Hedrich, Uncovering a Microbial Enigma: Isolation and Characterization of the Streamer-Generating, Iron-Oxidizing, Acidophilic Bacterium “Ferrovum myxofaciens.” *Appl. Environ. Microbiol.* **80**, 672–680 (2014).

19. V. V. Kadnikov, *et al.*, A novel uncultured bacterium of the family Gallionellaceae: Description and genome reconstruction based on metagenomic analysis of microbial community in acid mine drainage. *Microbiol. (Russian Fed.* **85**, 449–461 (2016).

20. K. and I. Hayashi, Ishida, Yokota, “Hydrogenophilus” in *Bergey’s Manual of Systematics of Archaea and Bacteria*, (Wiley, 2015), pp. 1–3.

21. A. Bollmann, *et al.*, Complete genome sequence of Nitrosomonas sp. Is79, an ammonia oxidizing bacterium adapted to low ammonium concentrations. *Stand. Genomic Sci.* **7**, 469–482 (2013).

22. H. Kojima, M. Fukui, Sulfuritalea hydrogenivorans gen. nov., sp. nov., a facultative autotroph isolated from a freshwater lake. *Int. J. Syst. Evol. Microbiol.* **61**, 1651–1655 (2011).

23. T. Watanabe, H. Kojima, M. Fukui, Sulfuriferula multivorans gen. nov., sp. nov., isolated from a freshwater lake, reclassification of ‘Thiobacillus plumbophilus’ as Sulfuriferula plumbophilus sp. nov., and description of Sulfuricellaceae fam. nov. and Sulfuricellales ord. nov. *Int. J. Syst. Evol. Microbiol.* **65**, 1504–1508 (2015).
